# Determination of the best multivariate adaptive geographically weighted generalized Poisson regression splines model employing generalized cross-validation in dengue fever cases

**DOI:** 10.1016/j.mex.2023.102174

**Published:** 2023-04-07

**Authors:** Riry Sriningsih, Bambang Widjanarko Otok, Sutikno

**Affiliations:** a Department of Statistics, Faculty of Science and Data Analytics, Institut Teknologi Sepuluh Nopember, Surabaya 60111, Indonesia; b Department of Mathematics, Faculty of Mathematics and Natural Sciences, Universitas Negeri Padang, West Sumatera, Indonesia

**Keywords:** DHF, GCV, Generalized Poisson, GWGPR, MARS, MAGPRS, MAGWGPRS, MSE, Weighted-MLE, Multivariate Adaptive Geographically Weighted Generalized Poisson Regression Splines (MAGWGPRS)

## Abstract

This article constructs a new model based on multivariate adaptive generalized Poisson regression splines (MAGPRS) and geographically weighted generalized Poisson regression (GWGPR), which is known as multivariate adaptive geographically weighted generalized Poisson regression splines (MAGWGPRS). The article elaborates the steps of weighted maximum likelihood estimation (weighted-MLE) to obtain the estimated values of its parameters. MAGWGPRS and MAGPRS were applied to the number of dengue hemorrhagic fever (DHF) cases in 119 districts or cities in Java, Indonesia, in 2020, to compare their performance. The fitted value plot versus actual data and a comparison of the mean square error (MSE) value demonstrate the goodness of the two models. The best MAGWGPRS model for each location was obtained, and only one the best MAGPRS model for all locations was acquired. Based on the plot results of the fitted value with the actual data and MSE value, MAGWGPRS is determined to be superior to MAGPRS.

## Review Highlights

• We present MAGWGPRS, a new model that is a combination and development of count response MARS and spatial regression.
• Selecting the optimal bandwidth using an adaptive Gaussian kernel function based on the CV method.
• Grouping districts/cities in Java Indonesia based on inter-regional disaggregating variables in modeling DHF cases.

## Graphical abstract

**Figure:**
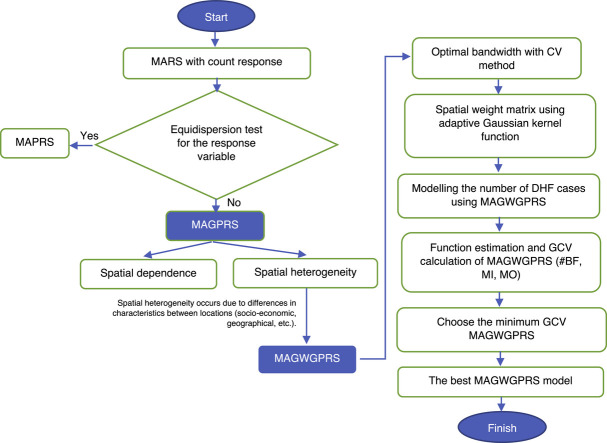

Specifications table

**Table:** 

Subject area:	Mathematics and Statistics

More specific subject area:	Statistics: Nonparametric Regression, Spatial Regression
Name of your method:	Multivariate Adaptive Geographically Weighted Generalized Poisson Regression Splines (MAGWGPRS)
Name and reference of original method:	Original Method Multivariate adaptive regression splines (MARS) Geographically weighted generalized Poisson regression (GWGPR) Reference A.P. Ampulembang, B.W. Otok, A.T. Rumiati, Budiasih, Bi-responses nonparametric regression model using MARS and its properties, Appl. Math. Sci. 9 (2015) 1417–1427. https://doi.org/10.12988/ams.2015.5127. S. Hidayati, B.W. Otok, Purhadi, Parameter Estimation and Statistical Test in Multivariate Adaptive Generalized Poisson Regression Splines, IOP Conf. Ser. Mater. Sci. Eng. 546 (2019) 1–11. https://doi.org/10.1088/1757–899X/546/5/052051. S.W. Tyas, L.A. Puspitasari, MethodsX Geographically weighted generalized poisson regression model with the best kernel function in the case of the number of postpartum maternal mortality in east java, MethodsX. 10 (2023) 102,002. https://doi.org/10.1016/j.mex.2023.102002.
Resource availability:	Dengue hemorrhagic fever (DHF) cases (y) and the predictors (x) from Badan Pusat Statistik (BPS) in each province in Java, Indonesia

## Method details

### Introduction

Multivariate adaptive regression splines (MARS) was first introduced by Friedman in 1991 [1]. Some studies that demonstrate the advantages of MARS compared to other regression models incorporate [2], [3], [4], [5]. Most MARS are developed for continuous [6], [7], [8], [9] and categorical responses [10], [11], [12], [13], however, MARS for count responses are still scarce and limited. MARS with count response is the result of combining MARS and Poisson regression, first discussed by Yasmirullah et al. (2021), which is MAPRS [14]. This model discusses the weighted least square (WLS) method for estimating model parameters. Hidayati et al. (2019) and Otok et al. (2019) combined MAPRS and generalized Poisson regression (GPR) into MAGPRS to solve the equidispersion problem [15,16].

Previous research has not considered the spatial variation precipitated by geographical location in the study area. In fact, there are many problems associated with spatial variation, such as a relationship between the response and predictor that varies depending on geographic location, and parameters that are not constant across the study area [17]. The spatial variation occurs because each location has different characteristics such as geographical, cultural, and socioeconomic differences. As a result, the same predictor variable can have different effects at different locations. One of the statistical techniques used to overcome spatial variation is geographically weighted Poisson regression (GWPR) by Collins (2010) and Nakaya et al. (2005) [17,18]. To overcome cases of overdispersion or underdispersion, Adryanta et al. (2019), Sabtika et al. (2021), and Tyas et al. (2023) developed the GWPR model into GWGPR [19], [20], [21].

The similarity between MAGPRS and GWGPR is that both are localized regressions. The basis functions of MAGPRS are local series which are employed to model complex (non-linear) relationships. As a result, the global model of MAGPRS is a weighted sum of the local models [1,22]. The locality of GWGPR is due to differences in characteristics between observation areas (spatial non-stationarity). As a result, each location has a spatial weight, and the parameter estimates of the resulting regression model will differ depending on its geographical location (latitude and longitude coordinates). The MAGPRS model is extended in this article by considering spatial variation between observation areas, a modification of MAGPRS [15,16] and GWGPR [19], [20], [21]. This development model is significantly different from the previous one. The structure of the model has changed significantly. The modified model causes the basis function and its parameters to be localized at the same time. As a result, the model form, algorithm, and analysis are more complex than in the previous model.

The performance of the proposed model was implemented to the number of DHF cases in Java, Indonesia in 2020. The research units are districts or cities in Java, Indonesia. DHF is an infectious disease caused by the dengue virus and transmitted by the bite of Aedes aegypti or albopicus mosquitoes. The host (human), the agent (virus), and the environment are all essential factors in the growth and spread of DHF, and they differ by region. As a result, DHF is spatial data related to geographical location [23], [24], [25], [26], [27], [28]. The spatial weight matrix is generated using an adaptive Gaussian kernel function, and the optimal bandwidth is determined using the cross-validation (CV) method. Finally, the best MAGWGPRS model is examined using the generalized cross-validation (GCV) method.

## Model specifications and estimation procedures

### MARS model

In summary, the MARS model can be elaborated as follows. Given for each response variable $y_{i}$, i=1,2,...,n and q predictor variables $x_{i}=\left(x_{i1},x_{i2},...,x_{iq}\right)$, where n is the number of observations, and suppose that the estimated function f satisfies the regression model

(1) $$y_{i}=f\left(x_{i}\right)+\varepsilon_{i},i=1,2,...,n,$$

where $\varepsilon_{i}$ is error random with mean 0 and variance $\sigma^{2}$.

Assume the function f in Eq. (1) is a linear combination of the basis functions $B_{m}\left(x_{i}\right),m=1,2,...,M$:

(2) $$f\left(x_{i}\right)=\gamma_{0}+\sum_{m=1}^{M}\gamma_{m}B_{m}\left(x_{i}\right),i=1,2,...,n,$$

with $\gamma_{0}$ is the coefficient of the parent basis function, $\gamma_{m}$ is the coefficient of the $m^{th}$ non-constant basis function. Each basis function is defined as a truncated spline function:

(3) $$B_{m}\left(x_{i}\right)=\prod_{k=1}^{K_{m}}{\left(s_{km}\left(x_{iv\left(k,m\right)}-t_{km}\right)\right)}_{+},$$

where $x_{iv(k,m)}\in {\left\{x_{j}\right\}}_{j=1}^{q}$, $t_{km}\in {\left\{x_{iv(k,m)}\right\}}_{i=1}^{n}$, the value of $s_{km}\in \left\{-1,+1\right\}$,

If $s_{km}=+1$, then $+{\left(x_{iv(k,m)}-t_{km}\right)}_{+}=\left\{ \begin{array}{c} x_{iv(k,m)}-t_{km};x_{iv(k,m)}>t_{km},\\ 0;x_{iv(k,m)}\leq t_{km}, \end{array}\right.$ and if $s_{km}=-1$, then $-{\left(x_{iv(k,m)}-t_{km}\right)}_{+}=\left\{ \begin{array}{c} t_{km}-x_{iv(k,m)};t_{km}>x_{iv(k,m)},\\ 0;t_{km}\leq x_{iv(k,m)}. \end{array}\right.$

The model of Eq. (1) where f is a function in Eq. (2) is identified the MARS model [1,9,29]. Here, $K_{m}$ is the degree of interaction, $s_{km}$ is the sign of the basis function at the $k^{th}$ interaction and the $m^{th}$ basis function*,* and $x_{iv(k,m)}$ is the $v^{th}$ predictor variable, in which *v* is the index of the predictor variable associated with the $k^{th}$ interaction and the $m^{th}$ basis function at the $i^{th}$ observation, $t_{km}$ is the knot value at the $k^{th}$ interaction and the $m^{th}$ basis function of the predictor variable $x_{iv(k,m)}$.

The MARS algorithm comprises of forward and backward stepwise [1,9,29]. Forward stepwise construct the MARS model by adding truncated spline basis functions (knots and interactions) until the model has the maximum number of basis functions. Following the completion of the forward stepwise process, a backward stepwise is performed to determine the number of feasible basis functions in the model. The basis function that contributes the least to the estimated response value based on the minimum GCV value is eliminated backward stepwise.

1. Forward Stepwise:
  a. Determine the parent basis function, i.e., $B_{0}=1$.
  b. Suppose there are M+1 basis functions, i.e., $B_{0},B_{1}\left(x_{i}\right),...,B_{M}\left(x_{i}\right)$, then add a new pair of basis functions:
    (4) $$B_{M+1}\left(x_{i}\right)=B_{m}^{*}\left(x_{i}\right){\left[+(x_{iv(k,m)}-t_{km})\right]}_{+}\;\text{and}$$
    (5) $$B_{M+2}\left(x_{i}\right)=B_{m}^{*}\left(x_{i}\right){\left[-(x_{iv(k,m)}-t_{km})\right]}_{+}.$$

The basis function $B_{m}^{*}\left(x_{i}\right)$ in Eq. (4) and Eq. (5) is the parent basis function, which is a member of the set of basis functions existing before the addition of the new basis function pair, $x_{iv(k,m)}$ is a predictor variable that does not exist in the parent basis function. The addition of new basis function pairs in the forward stepwise is carried out until the maximum number of basis functions is reached and is selected based on the minimum MSE.

2. Backward Stepwise. It is conducted after the maximum number of basis functions is obtained by forward stepwise.
  a. Selecting the forward basis functions one by one, with the exception of the parent basis function, and removing it if the GCV value decreases when the basis function is deleted.
  b. The deletion process is repeated until the GCV value does not decrease despite the fact that the remaining basis function is discarded. The optimal basis function is the basis function that remains after this backward stepwise procedure.

Friedman (1991) employed the GCV method in a backward stepwise procedure to select the optimal basis function in the MARS algorithm. The GCV method was developed from [30,31]

(6) $$GCV\left(M\right)=\frac{MSE}{{\left(1-\frac{C\left(\tilde{M}\right)}{n}\right)}^{2}}=\frac{\frac{1}{n}\sum_{i=1}^{n}{\left(y_{i}-{\hat{f}}_{M}\left(x_{i}\right)\right)}^{2}}{{\left(1-\frac{C\left(\tilde{M}\right)}{n}\right)}^{2}},$$

with

(7) $$C\left(\tilde{M}\right)=C\left(M\right)+\frac{dM}{2},$$

where $C(\tilde{M})$ is a complex function, C(M) is the number of parameters to be estimated, d is the degree of interaction, with the optimum value within the interval 2≤d≤4, and ${\hat{f}}_{M}\left(x_{i}\right)$ is the estimated value of the response variable on the basis function M and the $i^{th}$ observation.

### MAGPRS model

Given a Generalized Poisson (GP) probability function from [[32], [33], [34]]:

(8) $$P\left(y|\mu ,\phi \right)={\left(\frac{\mu }{1+\phi \mu }\right)}^{y}\left(\frac{{\left(1+\phi y\right)}^{y-1}}{y!}\right)\left(\exp\left(-\frac{\mu \left(1+\phi y\right)}{1+\phi \mu }\right)\right);\;y=0,1,2,...,$$

where μ is the mean of an event and ϕ (ϕ≠0) is the dispersion parameter.

If the response variable in Eq. (1) is GP distributed with the probability function given in Eq. (8), then the MAGPRS model:

(9) $$E\left(y_{i}\right)=\mu_{i}=\exp\left(\gamma_{0}+\sum_{m=1}^{M}\gamma_{m}\prod_{k=1}^{K_{m}}{\left(s_{\mathrm{km}}\left(x_{\mathrm{iv}\left(k,m\right)}-t_{\mathrm{km}}\right)\right)}_{+}\right),\;i=1,2,...,n,=\exp\left(\gamma_{0}+\sum_{m=1}^{M}\gamma_{m}B_{m}\left(x_{i}\right)\right)=\exp\left(b_{i}^{T}\gamma \right),\;i=1,2,...,n,$$

with

(10) $$\gamma ={\left(\gamma_{0},\gamma_{1},\ldots ,\gamma_{M}\right)}^{T}\;\;\text{and}\;b_{i}^{T}=\left(1,B_{1}\left(x_{i}\right),\ldots ,B_{M}\left(x_{i}\right)\right).$$

### GWGPR model

If $y_{i}∼GP\left(\mu \left(u_{i},v_{i}\right),\phi \left(u_{i},v_{i}\right)\right)$, i=1,2,…,n, and $\left(u_{i},v_{i}\right)$ is the coordinate point with $u_{i}$ is latitude and $v_{i}$ is the longitude at $i^{th}$location, then the GWGPR model can be written as follows [19,20].

(11) $$\mu \left(u_{i},v_{i}\right)=\exp\left(\beta_{0}\left(u_{i},v_{i}\right)+\sum_{j=1}^{q}\beta_{j}\left(u_{i},v_{i}\right)x_{\mathrm{ij}}\right),=\exp\left(x_{i}^{T}\beta \left(u_{i},v_{i}\right)\right),$$

with

$$\beta \left(u_{i},v_{i}\right)={\left(\beta_{0}\left(u_{i},v_{i}\right),\beta_{1}\left(u_{i},v_{i}\right),\ldots ,\beta_{q}\left(u_{i},v_{i}\right)\right)}^{T}\;\text{and}\;x_{i}^{T}=\left(1,x_{i1},\ldots ,x_{iq}\right).$$

$\beta_{0}\left(u_{i},v_{i}\right)$ is the intercept parameter at$i^{th}$location and $\beta_{j}\left(u_{i},v_{i}\right)$ are the parameters model for each $j^{th}$predictor variable at $i^{th}$location.

### MAGWGPRS model

Model Eq. (10) has global parameters but local basis functions, resulting in a single regression model for all observations (locations). As a result, we would like to create a model Eq. (10) that is location-dependent, particularly regarding MAGWGPRS, so that the new model has different parameters for each location. The geographical location at $i^{th}$ location, denoted $\left(u_{i},v_{i}\right)$, is defined as in GWGPR section.

Given $y_{i}∼GP\left(\mu \left(u_{i},v_{i}\right),\phi \left(u_{i},v_{i}\right)\right)$, i=1,2,…,n, then the MAGWGPRS model can be formed as follows

(12) $$\mu \left(u_{i},v_{i}\right)=\exp\left(b_{i}^{T}\gamma \left(u_{i},v_{i}\right)\right);\;i=1,2,...,n,$$

with

$$\begin{array}{ccc} b_{i}^{T} & = & \left(1,B_{1}\left(x_{i}\right),B_{2}\left(x_{i}\right),\cdots ,B_{M}\left(x_{i}\right)\right),\\ & = & \left(1,\prod_{k=1}^{K_{1}}{\left[s_{k1}\left(x_{\mathrm{iv}\left(k,1\right)}-t_{k1}\right)\right]}_{+},\prod_{k=1}^{K_{2}}{\left[s_{k2}\left(x_{\mathrm{iv}\left(k,2\right)}-t_{k2}\right)\right]}_{+},\cdots ,\prod_{k=1}^{K_{M}}{\left[s_{\mathrm{kM}}\left(x_{\mathrm{iv}\left(k,M\right)}-t_{\mathrm{kM}}\right)\right]}_{+}\right).\\ \gamma \left(u_{i},v_{i}\right) & = & {\left(\gamma_{0}\left(u_{i},v_{i}\right),\gamma_{1}\left(u_{i},v_{i}\right),\gamma_{2}\left(u_{i},v_{i}\right),\cdots ,\gamma_{M}\left(u_{i},v_{i}\right)\right)}^{T}, \end{array}$$

with$\gamma_{0}\left(u_{i},v_{i}\right)$ is the parameter of the parent basis function at $i^{th}$location and $\gamma_{m}\left(u_{i},v_{i}\right)$ is the parameter of the $m^{th}$ non-constant basis function at $i^{th}$location.

### Parameter estimation of the magwgprs model

The weighted MLE method estimates the MAGWGPRS model parameters at each location by assigning geographic weights. This method maximizes the log-likelihood function by solving a gradient function equal to zero [35]. The following theorem is provided to estimate the parameters of the MAGWGPRS model.

Theorem 1 *Given the MAGWGPRS model in*Eq. (12)*. When we use the weighted MLE method to calculate the model parameters, we obtain the parameter estimator equation, which is not closed form,* i.e.*,*

(13) $$\sum_{i^{*}=1}^{n}b_{i^{*}}w_{ii^{*}}y_{i^{*}}-\sum_{i^{*}=1}^{n}b_{i^{*}}w_{ii^{*}}\frac{\phi \left(u_{i},v_{i}\right)e^{b_{i^{*}}^{T}\hat{\gamma }\left(u_{i},v_{i}\right)}y_{i^{*}}}{1+\phi \left(u_{i},v_{i}\right)e^{b_{i^{*}}^{T}\hat{\gamma }\left(u_{i},v_{i}\right)}}-\sum_{i^{*}=1}^{n}b_{i^{*}}w_{ii^{*}}\frac{e^{b_{i^{*}}^{T}\hat{\gamma }\left(u_{i},v_{i}\right)}\left(1+\phi \left(u_{i},v_{i}\right)y_{i^{*}}\right)}{{\left(1+\phi \left(u_{i},v_{i}\right)e^{b_{i^{*}}^{T}\hat{\gamma }\left(u_{i},v_{i}\right)}\right)}^{2}}=0,$$

and

(14) $$-\sum_{i^{*}=1}^{n}w_{ii^{*}}y_{i^{*}}\frac{e^{b_{i^{*}}^{T}\gamma \left(u_{i},v_{i}\right)}}{1+\hat{\phi }\left(u_{i},v_{i}\right)e^{b_{i^{*}}^{T}\gamma \left(u_{i},v_{i}\right)}}+\sum_{i^{*}=1}^{n}w_{ii^{*}}\frac{y_{i^{*}}^{2}}{1+\hat{\phi }\left(u_{i},v_{i}\right)y_{i^{*}}}-\sum_{i^{*}=1}^{n}w_{ii^{*}}\frac{e^{b_{i^{*}}^{T}\gamma \left(u_{i},v_{i}\right)}\left(y_{i^{*}}-e^{b_{i^{*}}^{T}\gamma \left(u_{i},v_{i}\right)}\right)}{{\left(1+\hat{\phi }\left(u_{i},v_{i}\right)e^{b_{i^{*}}^{T}\gamma \left(u_{i},v_{i}\right)}\right)}^{2}}=0.$$

Proof of Theorem 1. Based on the probability function in Eq. (8), the likelihood function for MAGWGPRS model

(15) $$\begin{array}{ccc} & & L\left(\gamma \left(u_{i},v_{i}\right),\phi \left(u_{i},v_{i}\right);i=1,2,...,n\right)=\prod_{i=1}^{n}P\left(y_{i}|\gamma \left(u_{i},v_{i}\right),\phi \left(u_{i},v_{i}\right)\right),\\ & & \;=\prod_{i=1}^{n}\left({\left(\frac{e^{b_{i}^{T}\gamma \left(u_{i},v_{i}\right)}}{1+\phi \left(u_{i},v_{i}\right)e^{b_{i}^{T}\gamma \left(u_{i},v_{i}\right)}}\right)}^{y_{i}}\left(\frac{{\left(1+\phi \left(u_{i},v_{i}\right)y_{i}\right)}^{y_{i}-1}}{y_{i}!}\right)\left(\exp\left(\frac{-e^{b_{i}^{T}\gamma \left(u_{i},v_{i}\right)}\left(1+\phi \left(u_{i},v_{i}\right)y_{i}\right)}{1+\phi \left(u_{i},v_{i}\right)e^{b_{i}^{T}\gamma \left(u_{i},v_{i}\right)}}\right)\right)\right). \end{array}$$

The natural logarithm function of Eq. (15) is

(16) $$\begin{array}{ccc} & & \mathrm{lnL}\left(\gamma \left(u_{i},v_{i}\right),\phi \left(u_{i},v_{i}\right);i=1,2,...,n\right)=\sum_{i=1}^{n}\mathrm{lnP}\left(y_{i}|\gamma \left(u_{i},v_{i}\right),\phi \left(u_{i},v_{i}\right)\right),\\ & & =\sum_{i=1}^{n}b_{i}^{T}\gamma \left(u_{i},v_{i}\right)y_{i}-\sum_{i=1}^{n}y_{i}\ln\left(1+\phi \left(u_{i},v_{i}\right)e^{b_{i}^{T}\gamma \left(u_{i},v_{i}\right)}\right)+\sum_{i=1}^{n}\left(y_{i}-1\right)\ln\left(1+\phi \left(u_{i},v_{i}\right)y_{i}\right)+\\ & & -\sum_{i=1}^{n}\ln\left(y_{i}!\right)-\sum_{i=1}^{n}\frac{e^{b_{i}^{T}\gamma \left(u_{i},v_{i}\right)}\left(1+\phi \left(u_{i},v_{i}\right)y_{i}\right)}{1+\phi \left(u_{i},v_{i}\right)e^{b_{i}^{T}\gamma \left(u_{i},v_{i}\right)}}. \end{array}$$

To estimate the parameters of the MAGWGPRS model at $i^{th}$location requires spatial weighting. It utilizes distance information from one location to another. Let i=1,2,…,n represent the location in which the local parameter estimates are generated (i.e., regression points) and $i^{*}=1,2,\ldots ,n$ represent the location in which the data has been observed (i.e., observation points). The spatial weight,$w_{ii^{*}}$, represents the weight assigned to the $n^{th}$ observation in the calibration of the MAGWGPRS model for the $n^{th}$ location. This research employs the Gaussian kernel adaptive weighting function in [36], which is defined as $w_{ii^{*}}=\exp\left(-\frac{1}{2}{\left(\frac{d_{ii^{*}}}{h_{i}}\right)}^{2}\right)$ where $d_{ii^{*}}=\sqrt{{\left(u_{i}-u_{i^{*}}\right)}^{2}+{\left(v_{i}-v_{i^{*}}\right)}^{2}}$ is the Euclidean distance between the ${i^{*}}^{th}$ observation and the $i^{th}$ regression point and $h_{i}$ is the bandwidth at the $i^{th}$ regression point.

According to Nakaya et al. (2005), the bandwidth governs the rate at which the datum weight decreases as the distance between the observation location and the regression point increases. If the bandwidth value is very small, the variance increases, thus, the number of observations within radius $h_{i}$ will be small. If the bandwidth value is tremendously large (close to infinity), the variance decreases and the resulting weight $w_{ii^{*}}$ between locations approaches 1, hence the estimated parameters will be homogeneous, and the spatial model will be similar to the global regression model [18]. The CV method is employed in [36] to obtain the optimum bandwidth:

$$CV\left(h_{i}\right)=\sum_{i=1}^{n}{\left(y_{i}-{\hat{y}}_{\neq i}\left(h_{i}\right)\right)}^{2},$$

where ${\hat{y}}_{\neq i}\left(h_{i}\right)$ is the estimated value of the observation for bandwidth $h_{i}$ except at the $i^{th}$ location.

Next, input $w_{ii^{*}}$ into Eq. (16) based on [37]:

(17) $$\begin{array}{ccc} & & \ln L^{*}\left(\gamma \left(u_{i},v_{i}\right),\phi \left(u_{i},v_{i}\right)\right)=\sum_{i^{*}=1}^{n}w_{ii^{*}}\mathrm{lnP}\left(y_{i^{*}}|\gamma \left(u_{i},v_{i}\right),\phi \left(u_{i},v_{i}\right)\right),\\ & & \;\underset{i^{*}=1}{\overset{n}{=\sum }}b_{i^{*}}^{T}\gamma \left(u_{i},v_{i}\right)w_{ii^{*}}y_{i^{*}}-\sum_{i^{*}=1}^{n}w_{ii^{*}}y_{i^{*}}\ln\left(1+\phi \left(u_{i},v_{i}\right)e^{b_{i^{*}}^{T}\gamma \left(u_{i},v_{i}\right)}\right)\\ & & \;\;+\sum_{i^{*}=1}^{n}w_{ii^{*}}\left(y_{i^{*}}-1\right)\ln\left(1+\phi \left(u_{i},v_{i}\right)y_{i^{*}}\right)-\sum_{i^{*}=1}^{n}w_{ii^{*}}\ln\left(y_{i^{*}}!\right)-\sum_{i^{*}=1}^{n}w_{ii^{*}}\frac{e^{b_{i^{*}}^{T}\gamma \left(u_{i},v_{i}\right)}\left(1+\phi \left(u_{i},v_{i}\right)y_{i^{*}}\right)}{1+\phi \left(u_{i},v_{i}\right)e^{b_{i^{*}}^{T}\gamma \left(u_{i},v_{i}\right)}}. \end{array}$$

Eq. (17) is derived partially for each parameter and equalized to zero using the weighted MLE method. The description is as follow

The first partial derivative of Eq. (17) with respect to $\gamma^{T}\left(u_{i},v_{i}\right)$ gives

$$\begin{array}{ccc} \frac{\partial \ln L^{*}\left(\gamma \left(u_{i},v_{i}\right),\phi \left(u_{i},v_{i}\right)\right)}{\partial \gamma^{T}\left(u_{i},v_{i}\right)} & = & \sum_{i^{*}=1}^{n}b_{i^{*}}w_{ii^{*}}y_{i^{*}}-\sum_{i^{*}=1}^{n}b_{i^{*}}w_{ii^{*}}\frac{\phi \left(u_{i},v_{i}\right)e^{b_{i^{*}}^{T}\gamma \left(u_{i},v_{i}\right)}y_{i^{*}}}{1+\phi \left(u_{i},v_{i}\right)e^{b_{i^{*}}^{T}\gamma \left(u_{i},v_{i}\right)}}+\\ & & -\sum_{i^{*}=1}^{n}b_{i^{*}}w_{ii^{*}}\frac{e^{b_{i^{*}}^{T}\gamma \left(u_{i},v_{i}\right)}\left(1+\phi \left(u_{i},v_{i}\right)y_{i^{*}}\right)}{{\left(1+\phi \left(u_{i},v_{i}\right)e^{b_{i^{*}}^{T}\gamma \left(u_{i},v_{i}\right)}\right)}^{2}}, \end{array}$$

hence

(18) $$\sum_{i^{*}=1}^{n}b_{i^{*}}w_{ii^{*}}y_{i^{*}}-\sum_{i^{*}=1}^{n}b_{i^{*}}w_{ii^{*}}\frac{\phi \left(u_{i},v_{i}\right)e^{b_{i^{*}}^{T}\hat{\gamma }\left(u_{i},v_{i}\right)}y_{i^{*}}}{1+\phi \left(u_{i},v_{i}\right)e^{b_{i^{*}}^{T}\hat{\gamma }\left(u_{i},v_{i}\right)}}-\sum_{i^{*}=1}^{n}b_{i^{*}}w_{ii^{*}}\frac{e^{b_{i^{*}}^{T}\hat{\gamma }\left(u_{i},v_{i}\right)}\left(1+\phi \left(u_{i},v_{i}\right)y_{i^{*}}\right)}{{\left(1+\phi \left(u_{i},v_{i}\right)e^{b_{i^{*}}^{T}\hat{\gamma }\left(u_{i},v_{i}\right)}\right)}^{2}}=0.$$

Similarly, the first partial derivative of Eq. (17) with respect to $\phi (u_{i},v_{i})$ gives

(19) $$-\sum_{i^{*}=1}^{n}w_{ii^{*}}y_{i^{*}}\frac{e^{b_{i^{*}}^{T}\gamma \left(u_{i},v_{i}\right)}}{1+\hat{\phi }\left(u_{i},v_{i}\right)e^{b_{i^{*}}^{T}\gamma \left(u_{i},v_{i}\right)}}+\sum_{i^{*}=1}^{n}w_{ii^{*}}\frac{y_{i^{*}}^{2}}{1+\hat{\phi }\left(u_{i},v_{i}\right)y_{i^{*}}}-\sum_{i^{*}=1}^{n}w_{ii^{*}}\frac{e^{b_{i^{*}}^{T}\gamma \left(u_{i},v_{i}\right)}\left(y_{i^{*}}-e^{b_{i^{*}}^{T}\gamma \left(u_{i},v_{i}\right)}\right)}{{\left(1+\hat{\phi }\left(u_{i},v_{i}\right)e^{b_{i^{*}}^{T}\gamma \left(u_{i},v_{i}\right)}\right)}^{2}}=0.$$

Eq. (18) and Eq. (19) are not closed form, then Theorem 1 is proven. □

Since Eq. (18) and Eq. (19) are not closed-form, they are solved by numerical methods, namely the Berndt Hall Hausman (BHHH) method. The advantages of the BHHH method compared to other numerical methods for parameter estimation include its robustness to misspecification of the underlying distribution of the data, its simplicity, its convergence properties, and its computational efficiency. Suppose the parameters in Eq. (18) and Eq. (19) are written in the form of $\theta_{{}_{MAGWGPRS}}={\left(\gamma^{T}\left(u_{i},v_{i}\right),\phi \left(u_{i},v_{i}\right)\right)}^{T}$, then the BHHH iteration process stops when $∥{\hat{\theta }}_{\left(l^{*}+1\right)}-{\hat{\theta }}_{\left(l^{*}\right)}∥\leq \varepsilon^{*}$. The BHHH iteration equation is

$${\hat{\theta }}_{{\left(l+1\right)}_{MAGWGPRS}}={\hat{\theta }}_{{\left(l\right)}_{MAGWGPRS}}-H^{-1}\left({\hat{\theta }}_{{\left(l\right)}_{MAGWGPRS}}\right)g\left({\hat{\theta }}_{{\left(l\right)}_{MAGWGPRS}}\right),\;l=0,1,2,...,l^{*},$$

where

$$\begin{array}{ccc} & & {\hat{\theta }}_{{\left(0\right)}_{MAGWGPRS}}={\left({\hat{\gamma }}_{\left(0\right)}^{T}\left(u_{i},v_{i}\right),{\hat{\phi }}_{\left(0\right)}\left(u_{i},v_{i}\right)\right)}^{T},\\ & & g\left({\hat{\theta }}_{{\left(l\right)}_{MAGWGPRS}}\right)={{\left(\frac{\partial \ln L^{*}\left(\theta_{MAGWGPRS}\right)}{\partial \gamma^{T}\left(u_{i},v_{i}\right)},\frac{\partial \ln L^{*}\left(\theta_{MAGWGPRS}\right)}{\partial \phi \left(u_{i},v_{i}\right)}\right)}^{T}|}_{\theta_{\left(l\right)}={\hat{\theta }}_{\left(l\right)}},\\ & & H\left({\hat{\theta }}_{{\left(l\right)}_{MAGWGPRS}}\right)=-{\sum_{i=1}^{n}\left(k_{i}\left({\hat{\theta }}_{{\left(l\right)}_{MAGWGPRS}}\right)\right)\left(k_{i}\left({\hat{\theta }}_{{\left(l\right)}_{MAGWGPRS}}\right)\right)}^{T}. \end{array}$$

Next, calculate the modified GCV MARS for the combination of basis function (BF), maximum interaction (MI), and minimum observation (MO). Finally, select the best MAGWGPRS model based on the minimum value of the modified GCV MARS.

#### Steps in the research

Stages of research analysis:

1. Data exploration.
2. Equidispersion test of response variables.
3. Spatial heterogeneity test with Breusch-Pagan method.
4. Calculate the Euclidean distance between locations.
5. Determine the optimum bandwidth with the CV method.
6. Calculate the spatial weight matrix of the adaptive Gaussian kernel function.
7. Calculate the GCV of MAGWGPRS (# BF, MI, MO).
8. Select the minimum GCV value.
9. Obtain the best MAGWGPRS model.

#### Model application

The MAGWGPRS model was implemented to data on DHF cases in 119 districts or cities in Java, Indonesia. For each district or city in 2020, data was obtained from Badan Pusat Statistik (BPS) [38], [39], [40], [41], [42], [43]. The research variables consisted of one response variable and six predictor variables. The response variable is the number of DHF cases in 119 districts/cities. The predictor variables: $x_{1}$ is population density (ha/person), $x_{2}$ is the percentage of households that possess access to proper sanitation, $x_{3}$ is the percentage of households which own access to proper drinking water sources, $x_{4}$ is the percentage of poor population, $x_{5}$ is the ratio of medical personnel, and $x_{6}$ is the ratio of health centers.

**Fig. 1:**
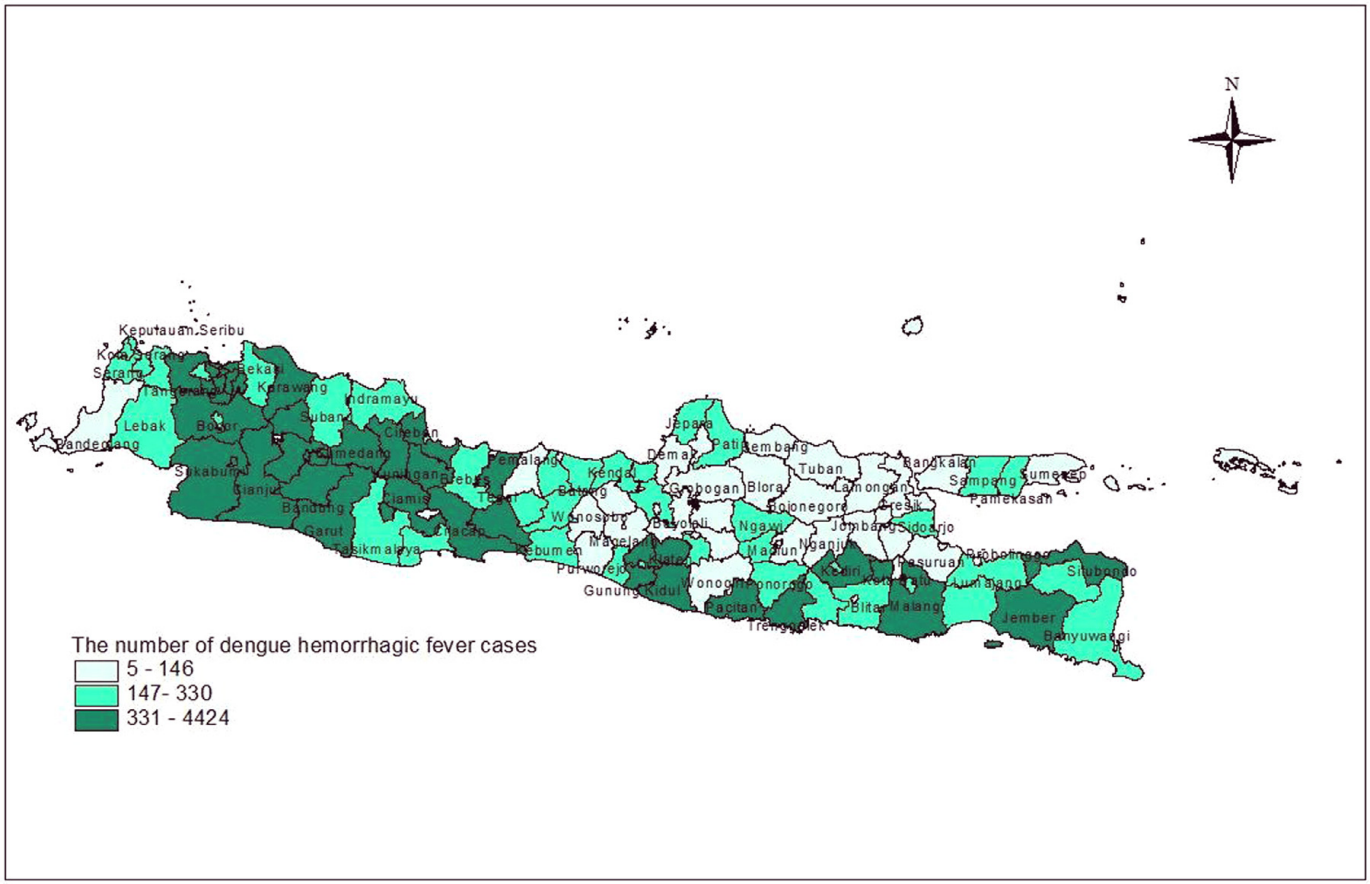
The distribution of DHF cases in districts/cities, Java Indonesia.

The map below depicts the distribution of DHF cases in Java, Indonesia. According to Fig. 1, the highest number of DHF cases were discovered in West Java Province (18 out of 27 districts/cities) and DKI Jakarta (4 out of 6 districts/cities).

To begin the analysis with MAGWGPRS, there are two steps performed. First, conduct an equidispersion test. According to [44], if the quotient of Pearson Chi-Square or deviance with free degrees is equal to one, the data is said to be equidispersion or (ϕ=1), overdispersion if ϕ>1, and underdispersion for other than that. In this research data, the deviance value is 34,807.71 with 112 free degrees, so the dispersion value is 310.783. This indicates that the data has overdispersion. Then, it is performed a spatial heterogeneity test. This test is employed to assess whether there are differences in characteristics between locations. The Breusch-Pagan (BP) test statistic is one that can be utilized. The BP value and p-value in this study are 19.996 and 0.002773, respectively. Because the p-value is less than 0.05, it can be concluded that spatial heterogeneity exists. As a result, the MAGWGPRS model has been validated for predicting the spread of DHF in 119 districts/cities in Java, Indonesia.

Next, estimate the parameters of the MAGWGPRS model for each location. The first step is to determine the optimum bandwidth, BF, MI, and MO. Based on the spatial weights corresponding to the location and the combination of BF, MI, and MO, 36 MAGWGPRS models are generated for each location. Then, for each location, select the best model from the 36 available models based on the smallest GCV or the largest $R^{2}$ value. The obtained results are 119 best MAGWGPRS models. Finally, the predictor variables are categorized as inter-regional disaggregates, that is, predictor variables that affect the model, as illustrated by Table 1.

**Table 1:** Grouping of districts/cities based on inter-regional disaggregating variables in the MAGWGPRS model.

Variables	Code of districts/cities^1^	Total of districts/cities
$x_{1},x_{2},x_{3},x_{4},x_{5},x_{6}$	1–26, 28–36, 40, 44–47, 49, 52, 59–62, 67, 68, 74–77, 86, 92–95, 98, 100, 103, 109, 112–119	69
$x_{1},x_{2},x_{3},x_{5},x_{6}$	27, 37–39, 57, 58, 78, 81, 87–90, 96, 97, 99, 101, 104–108, 110, 111	23
$x_{1},x_{2},x_{5},x_{6}$	41, 48, 51, 53–56, 63, 65, 66, 69–73, 79, 80, 83–85, 102	21
$x_{2},x_{3},x_{4},x_{5},x_{6}$	42, 43, 64	3
$x_{1},x_{2},x_{4},x_{5},x_{6}$	50, 82	2
$x_{2},x_{4},x_{5},x_{6}$	91	1

1 the districts/city code in Appendix A.

Table 1 demonstrates six groups of districts /cities based on the disaggregating variables between regions. District/city locations are symbolized by nonnegative integer codes, which can be perceived in Appendix A. For example, the groups of districts/cities where DHF cases are influenced by $x_{1},x_{2},x_{3},x_{4},x_{5},x_{6}$ are districts/cities 1–26, 28–36, 40, 44–47, 49, 52, 59–62, 67, 68, 74–77, 86, 92–95, 98, 100, 103, 109, 112–119. Districts in the same group therefore have the same MAGWGPRS model structure in the inter-regional disaggregating variables.

**Fig. 2:**
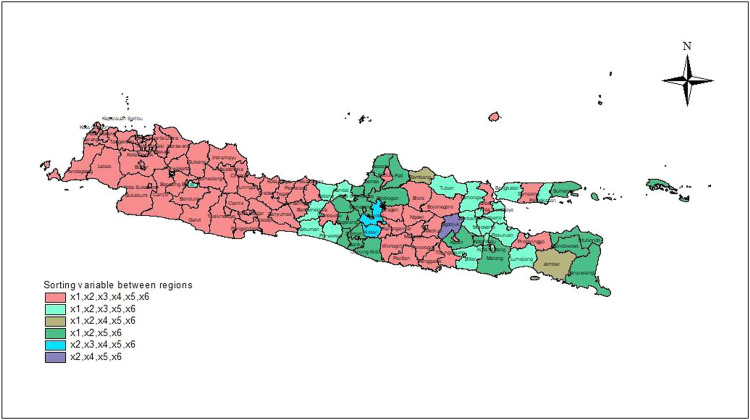
Grouping of districts/cities based on inter-regional disaggregating variables in the MAGWGPRS model.

Fig. 2 depicts the visual grouping of districts/cities based on the separating variables between regions.

Fig. 2 depicts six groups of districts with adjacent areas that have similar characteristics. The first district Nganjuk (code 91), where population density and the percentage of households with access to safe drinking water have no effect on the number of DHF cases. In two districts (codes 50 and 82), the percentage of households with access to a safe drinking water source has no effect on the number of DHF cases.

As an illustration, to obtain the MAGWGPRS model, we provide examples of Central Jakarta and Surabaya cities. The possible MAGWGPRS models for Central Jakarta and Surabaya cities in accordance with a combination of BF, MI, and MO are presented in Table 2. The best model parameter estimates are presented in Table 3 and Table 4, respectively.

**Table 2:** Combination of BF, MI, and MO models of MAGWGPRS in Central Jakarta and Surabaya cites.

Central Jakarta City					Surabaya City				
BF	MI	MO	GCV	R^2^	BF	MI	MO	GCV	R^2^
12	1	0	2.040	0.425	12	1	0	2.514	0.191
12	1	1	2.201	0.380	12	1	1	2.418	0.222
12	1	2	2.207	0.378	12	1	2	2.152	0.308
12	1	3	0.073	0.416	12	1	3	2.214	0.288
12	2	0	1.608	0.547	12	2	0	2.073	0.333
12	2	1	1.909	0.462	12	2	1	1.598	0.486
12	2	2	1.941	0.453	12	2	2	1.861	0.401
12	2	3	1.281	0.639	12	2	3	2.276	0.268
12	3	0	1.608	0.547	12	3	0	1.847	0.406
12	3	1	1.909	0.462	12	3	1	1.328	0.573
12	3	2	1.878	0.471	12	3	2	1.720	0.447
12	3	3	1.281	0.639	12	3	3	1.720	0.447
18	1	0	1.612	0.546	18	1	0	2.432	0.218
18	1	1	1.265	0.644	18	1	1	1.928	0.380
18	1	2	1.535	0.568	18	1	2	2.087	0.329
18	1	3	1.622	0.543	18	1	3	2.104	0.323
18	2	0	1.214	0.658	18	2	0	1.995	0.358
18	2	1	1.500	0.577	18	2	1	1.352	0.565
18	2	2	1.521	0.572	18	2	2	1.705	0.452
18	2	3	1.130	0.682	18	2	3	2.024	0.349
18	3	0	1.015	0.714	18	3	0	1.728	0.444
18	3	1	1.500	0.577	18	3	1	0.842	0.729
18	3	2	1.551	0.563	18	3	2	1.198	0.615
18	3	3	1.074	0.697	18	3	3	1.392	0.552
24	1	0	1.506	0.576	24	1	0	2.318	0.254
24	1	1	1.080	0.696	24	1	1	1.557	0.499
24	1	2	1.229	0.654	24	1	2	1.824	0.413
24	1	3	1.361	0.617	24	1	3	1.970	0.366
24	2	0	0.842	0.763	24	2	0	1.903	0.388
24	2	1	1.391	0.608	24	2	1	1.187	0.618
24	2	2	1.348	0.620	24	2	2	1.577	0.493
24	2	3	1.016	0.714	24	2	3	1.712	0.449
24	3	0	0.883	0.751	24	3	0	1.466	0.528
24	3	1	1.218	0.657	24*	3*	1*	0.751*	0.759*
24	3	2	1.302	0.633	24	3	2	0.984	0.684
24*	3*	3*	0.823*	0.768*	24	3	3	1.121	0.640

⁎ The best model.

**Table 3:** Parameter estimation for the best model of Central Jakarta City.

Coefficient	Estimation	Std. Error	T value	Pr(>|t|)
Intercept	7.0160	$7.580\times 10^{-16}$	$9.255\times 10^{15}$	0.00⁎⁎
bx_magwgprs[,−1] $h(x_{1}-67.8785)$	−0.0023	$1.665\times 10^{-17}$	$-1.383\times 10^{14}$	0.00⁎⁎
bx_magwgprs[,−1] $h(67.8785-x_{1})$	0.0158	$1.634\times 10^{-17}$	$9.671\times 10^{14}$	0.00⁎⁎
bx_magwgprs[,−1] $h\left(x_{1}-67.8785\right)*h\left(x_{2}-69.9\right)$	0.0014	$1.059\times 10^{-18}$	$1.302\times 10^{15}$	0.00⁎⁎
bx_magwgprs[,−1] $h\left(x_{1}-67.8785\right)*h\left(69.9-x_{2}\right)$	0.0021	$1.241\times 10^{-18}$	$1.700\times 10^{15}$	0.00⁎⁎
bx_magwgprs[,−1] $h(x_{6}-1.35921)$	−2.5000	$2.800\times 10^{-16}$	$-8.928\times 10^{15}$	0.00⁎⁎
bx_magwgprs[,−1] $h(x_{3}-95.89)$	−0.3475	$1.569\times 10^{-16}$	$-2.215\times 10^{15}$	0.00⁎⁎
bx_magwgprs[,−1] $h(95.89-x_{3})$	−0.1022	$6.037\times 10^{-17}$	$-1.694\times 10^{15}$	0.00⁎⁎
bx_magwgprs[,−1] $h\left(67.8785-x_{1}\right)*h\left(x_{4}-6.78\right)$	−0.0046	$1.625\times 10^{-18}$	$-2.840\times 10^{15}$	0.00⁎⁎
bx_magwgprs[,−1] $h\left(67.8785-x_{1}\right)*h\left(6.78-x_{4}\right)$	−0.0058	$5.728\times 10^{-18}$	$-1.013\times 10^{15}$	0.00⁎⁎
bx_magwgprs[,−1] $h\left(x_{1}-67.8785\right)*h\left(x_{2}-69.9\right)*h\left(x_{3}-99.49\right)$	−0.0009	$1.666\times 10^{-18}$	$-5.641\times 10^{14}$	0.00⁎⁎
bx_magwgprs[,−1] $h\left(x_{1}-67.8785\right)*h\left(x_{2}-69.9\right)*h\left(99.49-x_{3}\right)$	−0.0017	$1.144\times 10^{-18}$	$-1.514\times 10^{15}$	0.00⁎⁎
bx_magwgprs[,−1] $h(x_{5}-0.942835)$	0.1595	$2.580\times 10^{-16}$	$6.180\times 10^{14}$	0.00⁎⁎
bx_magwgprs[,−1] $h\left(67.8785-x_{1}\right)*h\left(x_{2}-68.25\right)$	−0.0014	$5.879\times 10^{-19}$	$-2.339\times 10^{15}$	0.00⁎⁎
bx_magwgprs[,−1] $h\left(67.8785-x_{1}\right)*h\left(68.25-x_{2}\right)$	−0.0002	$1.076\times 10^{-18}$	$-1.880\times 10^{14}$	0.00⁎⁎
bx_magwgprs[,−1] $h\left(67.8785-x_{1}\right)*h\left(x_{2}-67.27\right)*h\left(x_{4}-6.78\right)$	0.0002	$8.709\times 10^{-20}$	$2.165\times 10^{15}$	0.00⁎⁎
bx_magwgprs[,−1] $h\left(67.8785-x_{1}\right)*h\left(67.27-x_{2}\right)*h\left(x_{4}-6.78\right)$	0.0001	$1.675\times 10^{-19}$	$4.414\times 10^{14}$	0.00⁎⁎
bx_magwgprs[,−1] $h\left(67.8785-x_{1}\right)*h\left(x_{3}-92.81\right)*h\left(x_{4}-6.78\right)$	0.0001	$2.561\times 10^{-19}$	$5.704\times 10^{14}$	0.00⁎⁎
bx_magwgprs[,−1] $h\left(67.8785-x_{1}\right)*h\left(92.81-x_{3}\right)*h\left(x_{4}-6.78\right)$	0.0002	$2.561\times 10^{-19}$	$7.797\times 10^{14}$	0.00⁎⁎
bx_magwgprs[,−1] $h\left(x_{1}-67.8785\right)*h\left(x_{6}-0.708892\right)$	−0.0430	$2.509\times 10^{-17}$	$-1.716\times 10^{15}$	0.00⁎⁎

⁎⁎ Significant at 0.05.

**Table 4:** Parameter estimation for the best model of Surabaya City.

Coefficient	Estimation	Std. Error	T value	Pr(>|t|)
Intercept	4.8510	$7.298\times 10^{-16}$	$6.647\times 10^{15}$	0.00⁎⁎
bx_magwgprs[,−1] $h(102.668-x_{1})$	0.0091	$5.602\times 10^{-17}$	$1.632\times 10^{14}$	0.00⁎⁎
bx_magwgprs[,−1] $h(102.668-x_{1})$	−0.0023	$1.033\times 10^{-17}$	$-2.239\times 10^{14}$	0.00⁎⁎
bx_magwgprs[,−1] $h\left(x_{1}-102.668\right)*h\left(x_{2}-69.9\right)$	0.0008	$2.429\times 10^{-18}$	$3.215\times 10^{14}$	0.00⁎⁎
bx_magwgprs[,−1] $h\left(x_{1}-102.668\right)*h\left(69.9-x_{2}\right)$	0.0041	$3.574\times 10^{-18}$	$1.144\times 10^{15}$	0.00⁎⁎
bx_magwgprs[,−1] $h\left(102.668-x_{1}\right)*h\left(x_{6}-0.638427\right)$	0.0144	$1.726\times 10^{-17}$	$8.365\times 10^{14}$	0.00⁎⁎
bx_magwgprs[,−1] $h\left(102.668-x_{1}\right)*h\left(0.638427-x_{6}\right)$	0.2826	$1.498\times 10^{-16}$	$1.887\times 10^{15}$	0.00⁎⁎
bx_magwgprs[,−1] $h\left(102.668-x_{1}\right)*h\left(x_{3}-90.49\right)*h\left(0.638427-x_{6}\right)$	−0.0318	$2.011\times 10^{-17}$	$-1.583\times 10^{15}$	0.00⁎⁎
bx_magwgprs[,−1] $h\left(102.668-x_{1}\right)*h\left(90.49-x_{3}\right)*h\left(0.638427-x_{6}\right)$	−0.0230	$2.873\times 10^{-17}$	$-8.002\times 10^{14}$	0.00⁎⁎
bx_magwgprs[,−1] $h\left(102.668-x_{1}\right)*h\left(x_{2}-94.47\right)*h\left(x_{6}-0.638427\right)$	0.0146	$1.882\times 10^{-17}$	$7.743\times 10^{14}$	0.00⁎⁎
bx_magwgprs[,−1] $h\left(102.668-x_{1}\right)*h\left(94.47-x_{2}\right)*h\left(x_{6}-0.638427\right)$	−0.0001	$5.428\times 10^{-19}$	$-7.905\times 10^{13}$	0.00⁎⁎
bx_magwgprs[,−1] $h(x_{2}-97.96)$	−4.2800	$3.109\times 10^{-15}$	$-1.377\times 10^{15}$	0.00⁎⁎
bx_magwgprs[,−1] $h(97.96-x_{2})$	−0.0024	$7.620\times 10^{-17}$	$-3.183\times 10^{13}$	0.00⁎⁎
bx_magwgprs[,−1] $h\left(102.668-x_{1}\right)*h\left(x_{2}-96.9\right)$	0.0189	$1.987\times 10^{-17}$	$9.541\times 10^{14}$	0.00⁎⁎
bx_magwgprs[,−1] $h\left(102.668-x_{1}\right)*h\left(96.9-x_{2}\right)$	0.0009	$1.077\times 10^{-18}$	$8.048\times 10^{14}$	0.00⁎⁎
bx_magwgprs[,−1] $h\left(102.668-x_{1}\right)*h\left(96.9-x_{2}\right)*h\left(x_{5}-0.949508\right)$	−0.0001	$1.705\times 10^{-18}$	$-7.123\times 10^{13}$	0.00⁎⁎
bx_magwgprs[,−1] $h\left(102.668-x_{1}\right)*h\left(96.9-x_{2}\right)*h\left(0.949508-x_{5}\right)$	−0.0010	$6.335\times 10^{-19}$	$-1.561\times 10^{15}$	0.00⁎⁎
bx_magwgprs[,−1] $h\left(102.668-x_{1}\right)*h\left(x_{6}-1.20456\right)$	−0.0368	$4.078\times 10^{-17}$	$-9.016\times 10^{14}$	0.00⁎⁎
bx_magwgprs[,−1] $h(x_{6}-1.82238)$	0.3666	$2.005\times 10^{-15}$	$1.829\times 10^{14}$	0.00⁎⁎
bx_magwgprs[,−1] $h\left(102.668-x_{1}\right)*h\left(x_{3}-85.54\right)*h\left(x_{6}-1.20456\right)$	−0.0013	$3.584\times 10^{-18}$	$-3.617\times 10^{14}$	0.00⁎⁎

⁎⁎ Significant at 0.05.

Based on Table 2, the best model of Central Jakarta city is discovered in the 36th model with BF, MI, MO, GCV, and R^2^ are 24, 3, 3, 0.823, and 0.768 successively. Table 3 implies the parameter estimation of the best model for Central Jakarta with the inter-region disaggregating variables x_1_, x_2_, x_3_, x_4_, x_5_, and x_6_. For Surabaya city, the best model is the 34th model with BF, MI, MO, GCV, and R^2^ are 24, 3, 1, 0.751, and 0.759 respectively. From Table 4, the separating variables between regions that possess an effect are x_1_, x_2_, x_3_, x_5_, and x_6_, while x_4_ has no effect. As a result, all predictor variables have an impact on the number of DHF cases in Central Jakarta but not in Surabaya. The following are the best MAGWGPRS models for Central Jakarta and Surabaya respectively.

(20) $$\begin{array}{ccc} \ln\hat{\mu }\left(u_{4},v_{4}\right) & = & 7.0156+0.0158*BF_{1}-0.0023*BF_{2}-0.1022*BF_{3}-0.3475*BF_{4}+\\ & & 0.1595*BF_{5}-2.5000*BF_{6}-0.0014*BF_{1}*BF_{7}-0.0002*BF_{1}*BF_{8}+\\ & & 0.0014*BF_{2}*BF_{9}+0.0021*BF_{2}*BF_{10}-0.0046*BF_{1}*BF_{11}+\\ & & -0.0058*BF_{1}*BF_{12}-\;0.0430*BF_{2}*BF_{13}-0.0009*BF_{2}*BF_{9}*BF_{14}+\\ & & -0.0017*BF_{2}*BF_{9}*BF_{15}+\;0.0002*BF_{1}*BF_{16}*BF_{11}+\\ & & 0.0001*BF_{1}*BF_{17}*BF_{11}+0.0001*BF_{1}*BF_{18}*BF_{11}+\;0.0002*BF_{1}*BF_{19}*BF_{11}, \end{array}$$

where

$$\begin{array}{ccc} BF_{1} & = & h\left(67.8785-x_{1}\right),BF_{2}=h\left(x_{1}-67.8785\right),BF_{3}=h\left(95.89-x_{3}\right),\\ BF_{4} & = & h\left(x_{3}-95.89\right),BF_{5}=h\left(x_{5}-0.942835\right),BF_{6}=h\left(x_{6}-1.35921\right),\\ BF_{7} & = & h\left(x_{2}-68.25\right),BF_{8}=h\left(68.25-x_{2}\right),BF_{9}=h\left(x_{2}-69.9\right),\\ BF_{10} & = & h\left(69.9-x_{2}\right),BF_{11}=h\left(x_{4}-6.78\right),BF_{12}=h\left(6.78-x_{4}\right),\\ BF_{13} & = & h\left(x_{6}-0.708892\right),BF_{14}=h\left(x_{3}-99.49\right),BF_{15}=h\left(99.49-x_{3}\right),\\ BF_{16} & = & h\left(x_{2}-67.27\right),BF_{17}=h\left(67.27-x_{2}\right),BF_{18}=h\left(x_{3}-92.81\right),BF_{19}=h\left(92.81-x_{3}\right), \end{array}$$

and

(21) $$\begin{array}{ccc} \ln\hat{\mu }\left(u_{110},v_{110}\right) & = & 4.8508-0.0023*BF_{1}+0.0091*BF_{2}-0.0024*BF_{3}-4.2805*BF_{4}+\\ & & 0.3666*BF_{5}+0.0190*BF_{1}*BF_{6}+0.0009*BF_{1}*BF_{7}+\\ & & 0.0008*BF_{2}*BF_{8}+0.0041*BF_{2}*BF_{9}+0.0144*BF_{1}*BF_{10}+\\ & & 0.2826*BF_{1}*BF_{11}-0.0368*BF_{1}*BF_{12}-0.0001*BF_{1}*BF_{7}*F_{13}+\\ & & -\;0.0010*BF_{1}*BF_{7}*BF_{14}+0.0146*BF_{1}*BF_{15}*BF_{10}+\\ & & -\;0.0001*BF_{1}*BF_{16}*BF_{10}-0.0318*BF_{1}*BF_{17}*BF_{11}+\\ & & -\;0.0230*BF_{1}*BF_{18}*BF_{11}-0.0013*BF_{1}*BF_{19}*BF_{12}, \end{array}$$

where

(22) $$\begin{array}{ccc} BF_{1} & = & h\left(102.668-x_{1}\right),BF_{2}\;=h\left(x_{1}-102.668\right),BF_{3}\;=h\left(97.96-x_{2}\right),\\ BF_{4} & = & h\left(x_{2}-97.96\right),BF_{5}\;=h\left(x_{6}-1.82238\right),BF_{6}\;=h\left(x_{2}-96.9\right),\\ BF_{7} & = & h\left(96.9-x_{2}\right),BF_{8}\;=h\left(x_{2}-69.9\right),BF_{9}\;=h\left(69.9-x_{2}\right),\\ BF_{10} & = & h\left(x_{6}-0.638427\right),BF_{11}\;=h\left(0.638427-x_{6}\right),BF_{12}=\;h\left(x_{6}-1.20456\right),\\ BF_{13} & = & \;h\left(x_{5}-0.949508\right),BF_{14}\;=h\left(0.949508-x_{5}\right),BF_{15}\;=h\left(x_{2}-94.47\right),\\ BF_{16} & = & h\left(94.47-x_{2}\right),BF_{17}=\;h\left(x_{3}-90.49\right),BF_{18}\;=h\left(90.49-x_{3}\right),BF_{19}=\;h\left(x_{3}-85.54\right). \end{array}$$

**Fig. 3:**
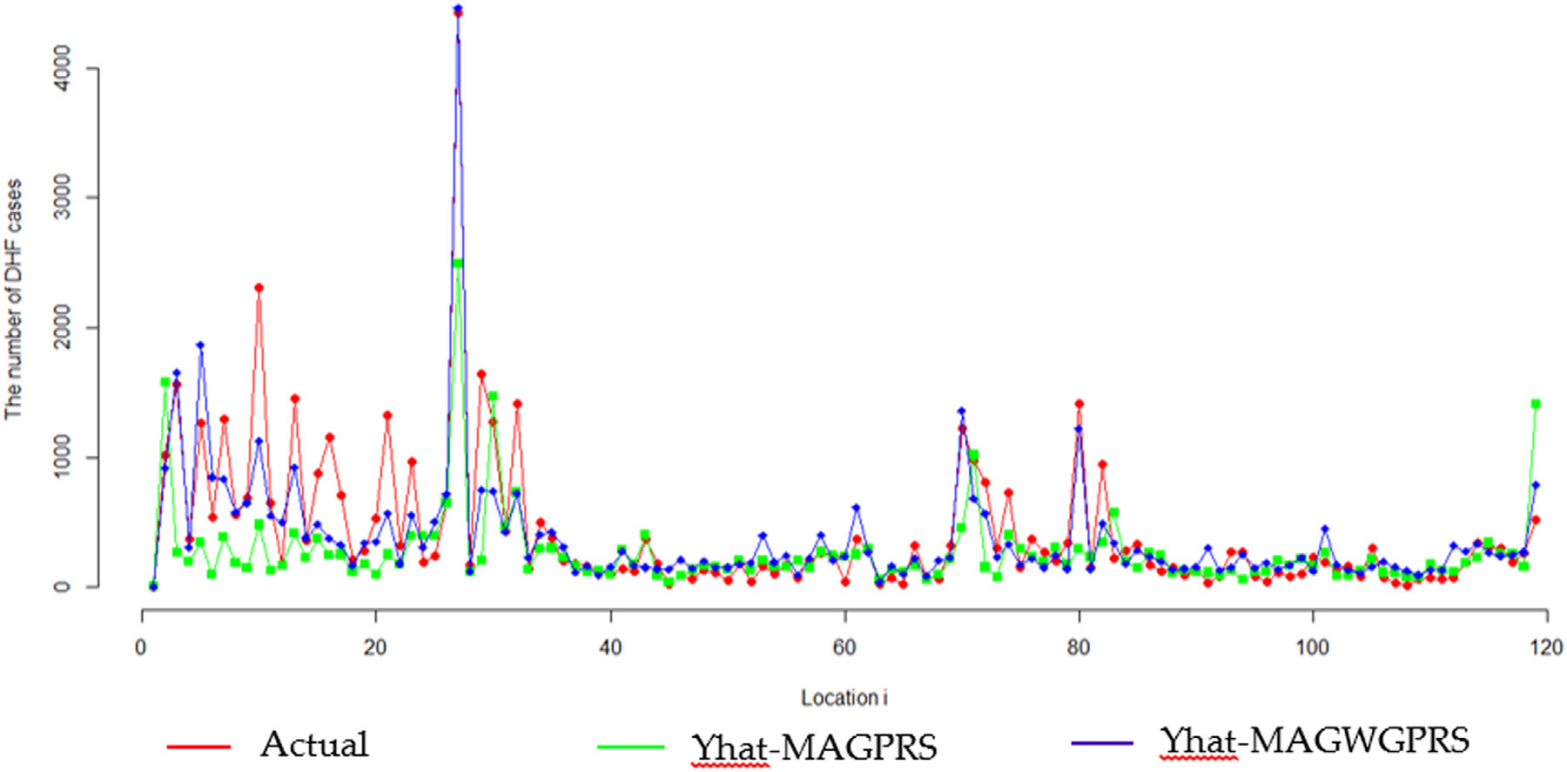
Comparison of the actual value and the fitted value of the response variable in the MAGPRS and MAGWGPRS models.

Furthermore, the MAGPRS model was compared to the performance of the best MAGWGPRS model for Central Jakarta city, Surabaya city, and 117 other districts/cities. Comparisons were made between the actual number of DHF cases and the fitted values of the best MAGWGPRS and MAGPRS models. The MSE value of each model may also be employed to compare the goodness of the two models. Fig. 3 illustrates the data plot of the actual number of DHF cases, the fitted value of the MAGWGPRS model, and the fitted value of the MAGPRS model. In general, the MAGWGPRS fitted values are closer to the actual values than the MAGPRS fitted values. According to the graph, the MAGWGPRS curve pattern is more similar to and closer to the actual curve pattern than the MAGPRS curve pattern. The MSE value of the MAGWGPRS model is less than the MSE value of the MAGPRS model, as illustrated by Table 5.

**Table 5:** Comparison of MSE values for the MAGPRS and MAGWGPRS.

Model	MSE
MAGPRS	190,817
MAGWGPRS	62,157

## Conclusion

The MAGWGPRS model, a modification of the MAGPRS model and GWGPR spatial regression, was proposed in this study. We demonstrated a step-by-step procedure for obtaining the estimated parameters of the MAGWGPRS model's estimated parameters. Aside from the benefit of displaying the regression coefficients for each location, MAGWGPRS is a complex model that necessitates a difficult coding program. As a result, determining the best model form becomes more complicated than the MAGPRS model.

Furthermore, the MAGWGPRS model was applied to dengue case data in 119 districts or cities on the Indonesian island of Java. The best model was obtained for each location based on the optimal bandwidth and spatial weighting with adaptive Gaussian kernel and the combination of BF, MI, and MO, specifically providing examples for the Central Jakarta and Surabaya cities. There are 119 best models, but if we employ the MAGPRS model to analyze them, we only obtain one of the best models for all locations. Based on the graphical comparison of actual values with MAGPRS and MAGWGPRS fit values and MSE values, the MAGWGPRS model is better than the MAGPRS model in this case.

The limitation of this article is that there is no hypothesis testing of the proposed model. Therefore, future research can test hypotheses. Furthermore, for various case studies, this model can be extended with other distributions or kernel functions.

## Ethics statements

The data used in this research are secondary data derived from the official website of BPS Provinces in Java, Indonesia.

## CRediT author statement

**Riry Sriningsih:** Conceptualization, methodology, and writing-preparation of the first draft. **Bambang Widjanarko Otok:** Conceptualization, methodology, writing-reviewing and editing, and supervision. **Sutikno:** Conceptualization, methodology, writing-reviewing and editing, and supervision.

## Declaration of Competing Interest

The authors declare that they have no known competing financial interests or personal relationships that could have appeared to influence the work reported in this paper.

## Figures and Tables

**Table A1:** Codes and names of districts/cities on the island of Java, Indonesia.

Code and Name	Code and Name	Code and Name	Code and Name
1. The Thousand Islands	31.Cimahi City	61. Tegal	91. Nganjuk
2. South Jakarta City	32.Tasikmalaya City	62. Brebes	92. Madiun
3. East Jakarta City	33. Banjar City	63. Magelang City	93. Magetan
4. Central Jakarta City	34. Cilacap	64. Surakarta City	94. Ngawi
5. West Jakarta City	35. Banyumas	65. Salatiga City	95. Bojonegoro
6. North Jakarta City	36. Purbalingga	66. Semarang City	96. Tuban
7. Bogor	37. Banjarnegara	67. Pekalongan City	97. Lamongan
8. Sukabumi	38. Kebumen	68. Tegal City	98. Gresik
9. Cianjur	39. Purworejo	69. Kulonprogo	99. Bangkalan
10. Bandung	40. Wonosobo	70. Bantul	100. Sampang
11. Garut	41. Magelang	71. Gunung Kidul	101. Pamekasan
12. Tasikmalaya	42. Boyolali	72. Sleman	102. Sumenep
13. Ciamis	43. Klaten	73. Yogyakarta City	103. Kediri City
14. Kuningan	44. Sukoharjo	74. Pacitan	104. Blitar City
15. Cirebon	45. Wonogiri	75. Ponorogo	105. Malang City
16. Majalengka	46. Karanganyar	76. Trenggalek	106. Probolinggo City
17. Sumedang	47. Sragen	77. Tulungagung	107. Pasuruan City
18. Indramayu	48. Grobogan	78. Blitar	108. Mojokerto City
19. Subang	49. Blora	79. Kediri	109. Madiun City
20. Purwakarta	50. Rembang	80. Malang	110. Surabaya City
21. Karawang	51. Pati	81. Lumajang	111. Batu City
22. Bekasi	52. Kudus	82. Jember	112. Pandeglang
23. West Bandung	53. Jepara	83. Banyuwangi	113. Lebak
24. Pangandaran	54. Demak	84. Bondowoso	114. Tangerang
25. Bogor City	55. Semarang	85. Situbondo	115. Serang
26. Sukabumi City	56. Temanggung	86. Probolinggo	116. Tangerang City
27. Bandung City	57. Kendal	87. Pasuruan	117. Cilegon City
28. Cirebon City	58. Batang	88. Sidoarjo	118. Serang City
29. Bekasi City	59. Pekalongan	89. Mojokerto	119. South Tangerang City
30. Depok City	60. Pemalang	90. Jombang	

## Data Availability

The authors do not have permission to share data.

## Appendix A

Table A1 shows the codes and names of districts/cities used and analyzed in the study.
